# Global trends in antimicrobial resistance on organic and conventional farms

**DOI:** 10.1038/s41598-023-47862-7

**Published:** 2023-12-18

**Authors:** Eldon O. Ager, Tamilie Carvalho, Erin M. Silva, Steven C. Ricke, Jessica L. Hite

**Affiliations:** 1 Department of Integrative Biology, University of Wisconsin, Madison, WI USA; 2 The Royal (Dick) School of Veterinary Studies and the Roslin Institute, University of Edinburgh, Midlothian, UK; 3 Department of Ecology and Evolutionary Biology, University of Michigan, Ann Arbor, MI 48109 USA; 4 Center for Integrated Agricultural Systems, University of Wisconsin, Madison, WI USA; 5 Department of Animal and Dairy Sciences, University of Wisconsin, Madison, WI USA; 6 Department of Pathobiological Sciences, University of Wisconsin, Madison, WI USA

**Keywords:** Antimicrobials, Bacteriology

## Abstract

The important hypothesis that organic livestock management reduces the prevalence of antimicrobial resistance is either fiercely supported or bitterly contested. Yet, empirical evidence supporting this view remains fragmentary, in part because relationships between antimicrobial use and drug resistance vary dramatically across contexts, hosts, pathogens, and country-specific regulations. Here, we synthesize global policies and definitions of ‘organic’ and ask if organic farming results in notable reductions in the prevalence of antimicrobial resistance when directly examined alongside conventional analogs. We synthesized the results of 72 studies, spanning 22 countries and five pathogens. Our results highlight substantial variations in country-specific policies on drug use and definitions of ‘organic’ that hinder broad-scale and generalizable patterns. Overall, conventional farms had slightly higher levels of antimicrobial resistance (28%) relative to organic counterparts (18%), although we found significant context-dependent variation in this pattern. Notably, environmental samples from organic and conventional farms often exhibited high levels of resistance to medically important drugs, underscoring the need for more stringent and consistent policies to control antimicrobial contaminants in the soil (particularly on organic farms, where the application of conventional manure could faciliate the spread antimicrobial resistance). Taken together, these results emphasize the challenges inherent in understanding links between drug use and drug resistance, the critical need for global standards governing organic policies, and greater investment in viable alternatives for managing disease in livestock.

## Introduction

Antimicrobial resistance (AMR) is an increasingly urgent global health crisis that disproportionately affects low- and middle-income countries^1,2^. The use (and overuse) of antimicrobials in livestock is generally linked with the rise of drug-resistant infections — in both animals and humans^3,4^. Globally, the majority (~ 73%) of all antibiotics are used in animals raised for food^5^, with over 45 mg/population correction unit (PCU) in cattle, 148 mg/PCU, and 172 mg/PCU in chickens and pigs, respectively^6^. A key objective, therefore, is to understand how the use of antimicrobials in livestock shapes the prevalence, identity, and transmission of antimicrobial resistance. Answers to this central, but challenging, question govern best practices for policy, food safety, and public health.

‘Organic’ and ‘non-conventional’ farming practices are proffered as a means to reduce the use of antimicrobials in livestock, therefore reducing the selective pressures that promote the evolution of drug resistance^7,8^. Indeed, mounting evidence suggests that organic farming practices can reduce the occurrence of pathogenic outbreaks and the presence of genes that carry and spread antimicrobial resistance^9–11^. These patterns offer a potential win–win solution for animal welfare, conservation, and public health. Mechanistic insight into how, when, and where organic farming practices substantially and reliably reduce the prevalence and spread of antimicrobial resistance could be used to formulate scalable solutions for conventional farming practices with benefits for both agriculture and public health. However, our understanding of the extent to which organic farming practices can successfully reduce the emergence and spread of AMR remains fragmentary — and hotly debated^12–15^. Empirical evidence linking specific farming practices to patterns of antimicrobial resistance remains difficult to establish, in part because the relationship between antimicrobial use and drug resistance varies dramatically across contexts, depending on environmental conditions, livestock hosts, and pathogens. Additionally, pronounced differences in country-specific regulations undermine efforts to develop universal standards, exhaust consumer confidence, and weaken economic efficiency. These critical gaps in knowledge represent a major impediment to policy and management decisions and hamper investment in the research and development needed to generate viable and scalable non-pharmacological alternatives for managing disease in livestock.

As a first step in addressing these concerns, we begin by synthesizing global differences in policies and definitions of ‘organic.’ Broadly speaking, conventional livestock production focuses on technologies for increased productivity, such as high-yielding breeds, modern feeding techniques and veterinary health products, as well as (synthetic) fertilizers and pesticides. In contrast, organic practices focus on reducing antimicrobial use in livestock by integrating cultural, biological, nutritional, and mechanical methods to ensure environmentally safe and residue-free foods, along with improved animal welfare standards^16–19^. In general, organic production strives to provide animals with a more spacious and enriched environment, access to an outdoor range, and limited group sizes, all of which ostensibly improve animal health and reduce the need for medications, including antimicrobials. However, a central obstacle to identifying the more specific broad-scale and generalizable differences between ‘organic’ and ‘conventional’ farming practices resides in the lack of a global or even regional consensus about standard practices.

After synthesizing current differences between organic and conventional farming practices (Supplementary Table S1), we conduct a literature review to examine where and when organic farming results in notable reductions in the prevalence of antimicrobial resistance over the range of contexts in which it has been examined alongside conventional analogs.

## Results

**Figure 1:**
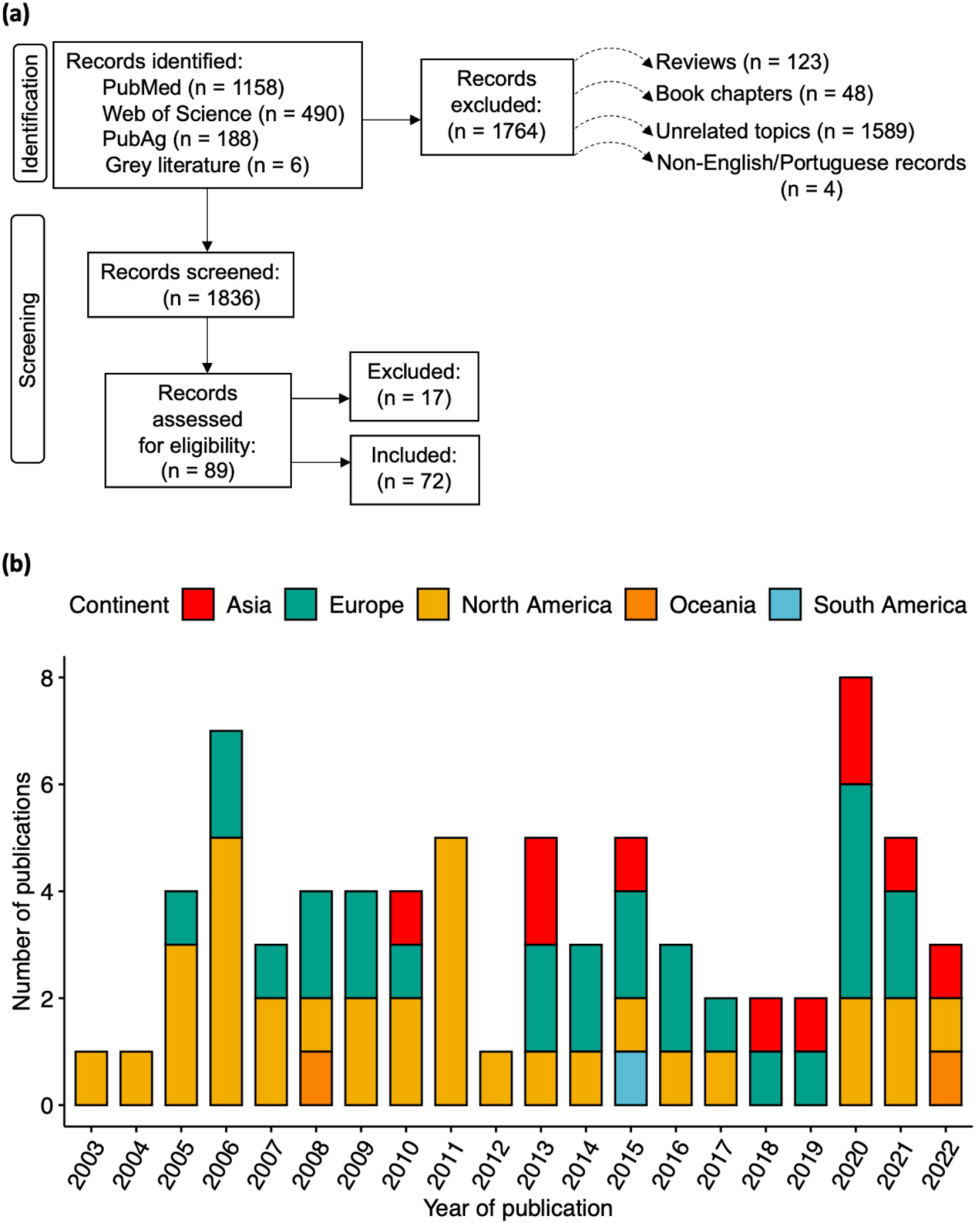
(**a**) Overview of PRISMA-based literature search results and categorization of studies. (**b**) After literature search and screening, 72 studies satisfied our inclusion criteria. These studies examined 109 farms and 61,299 bacterial isolates. The vast majority (46%) of these studies were in North America (*n* = 33), 36% were in Europe (*n*  = 26), 14% were in Asia (*n*  = 10), while Oceania and South America contributed to 3% (*n*  = 2) and 1% (*n*  = 1), respectively.

We identified 1,833 unique studies published between 2000 and 2022, as well as six grey literature studies (e.g., WHO website, with reports on point prevalence of antimicrobial resistance). After the references were screened, 1,744 studies and all six grey literature publications were removed (Fig. 1a). After assessing the remaining 89 references for eligibility, 17 studies were excluded, leaving 72 studies that met the inclusion criteria: 46% (*n* = 33). These studies spanned North America (46%*, n* = 33), Europe (36%, *n* = 26), Asia (14%, *n* = 10), Oceania (3%, *n* = 2) and South America (1%, *n* = 1) (Fig. 1b). All the surveys covered antimicrobial classifications based on the World Health Organization, and classifications including those considered ‘critically important' and ‘highly important' for human medicine. For all drug acronyms, see Supplementary Table S2. We first discuss overall global patterns, which largely arise due to the pronounced differences in region-specific regulations for the use of antimicrobials in livestock and variation in ‘organic' classification and certification standards (Supplementary Table S1). Then, we highlight key differences across host- region- and pathogen-specific contexts.

### Overall trends in AMR across organic and conventional farms

**Figure 2:**
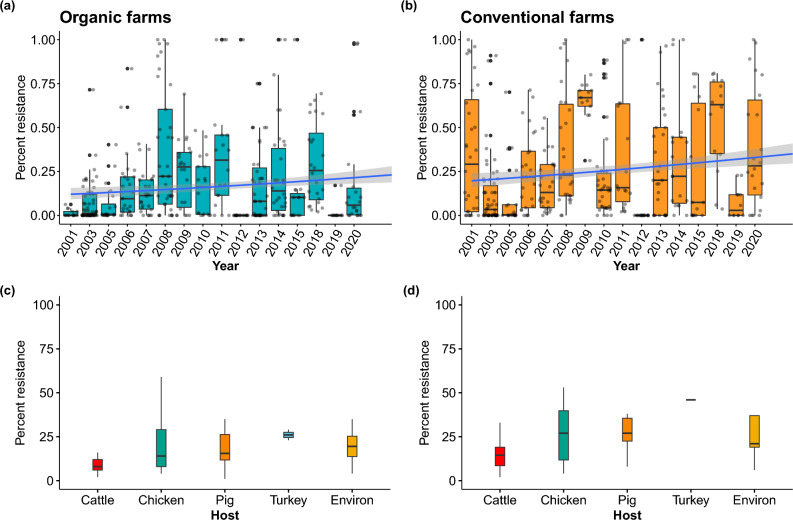
Temporal trends in the prevalence of antimicrobial resistance from different hosts (including environment samples) on organic and conventional farms. Note: 12 studies were excluded from this analysis because they did not report the year that the data were collected. Sixty surveys with complete sampling dates were included in the analysis. Globally, the prevalence of antimicrobial resistance was slightly lower on (**a**) organic farms (18%, *n* = 56) relative to conventional farms (28%, *n* = 53). However, antimicrobial resistance appears to be increasing on both (**c**) organic and (**d**) conventional farms. From 2001 to 2020, the prevalence of antimicrobial resistance in isolates from organic farms (*n* = 29,417) increased from 10% (CI: 8–13%) to 24% (CI: 20–28%), while the prevalence of drug resistance in isolates from conventional farms (*n* = 31,882) increased from 18% (CI: 15–23%) to 37% (CI: 32–43%). Examining host-specific patterns indicates that the prevalence of antimicrobial resistance from cattle, chicken, pig, and turkey isolates was higher on conventional farms as compared to organic farms. However, antimicrobial resistance was slightly higher in environmental samples collected from organic farms compared to conventional farms. Data represent the median $\pm$ the first and third quartile ranges.

Overall, the prevalence of antimicrobial resistance was higher on conventional farms (28%) relative to organic farms (18%), (Binomial GLM main effect of farm type (GLM), $\chi 2$ = 3,403, df = 1,* p* < 0.0001). However, between 2001 and 2020, antimicrobial resistance increased on both organic and conventional farms. AMR on organic farms increased from 10% (CI: 8–13%) to 24% (CI: 20–28%), while on conventional farms AMR increased from 18% (CI: 15–23%) to 37% (CI: 32–43%, $\chi 2$ = 15,429, df = 17,* p* < 0.0001, Fig. 2a and b).

### Host-specific patterns

Across all geographic regions, resistance patterns were highly variable among different host classes, with overall resistance higher in isolates from conventional farms. Conventional farms reported a higher AMR prevalence in isolates from cattle, chicken, pigs, and turkey. For instance, in isolates from cattle, the prevalence of AMR was 14.5% on conventional farms and 9% on organic farms (Fig. 2c and d). For chicken isolates, AMR prevalence was higher on conventional farms (22%) compared to organic farms (13.5%). Similar trends were reported for other hosts (Fig. 2c and d). On conventional pig farms 24.5% of isolates were resistant whereas on organic farms, only 15% of isolates were resistant. On conventional turkey farms, the prevalence of AMR was 46% as compared to 22.5% on organic farms. For environmental samples, the *median* prevalence of AMR in environmental isolates was slightly higher on organic farms, 16% relative to conventional farms, 11.5% (Fig. 2c and d). These patterns, however, were also highly variable across geographical regions.

### AMR patterns across broad geographic regions

**Figure 3:**
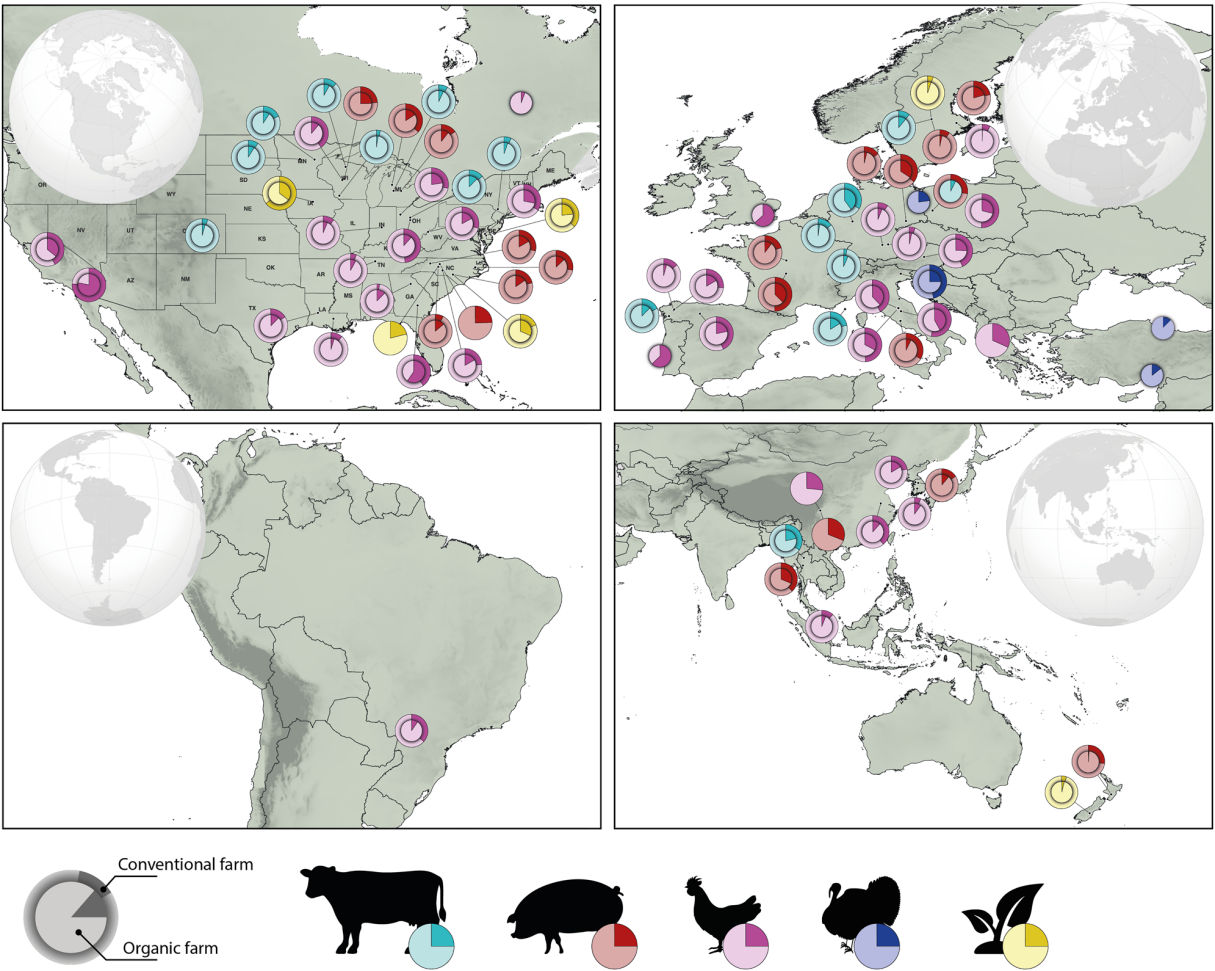
Global patterns of antimicrobial resistance in isolates collected from organic and conventional farms. Studies spanned four hosts (cattle, pig, chicken, and turkey) and environmental samples collected from conventional and organic farms throughout North America, Europe, Asia, Oceania, and South America. Pie charts show the prevalence of antimicrobial resistance on conventional farms (outer pie chart, *n*  = 66) relative to their organic counterparts (inner pie chart, *n*  = 69). Geographic regions with a single pie (i.e., outer pie only) represent areas lacking data from organic farms. The map (Fig. 3) was generated with QGIS version 3.24.0-Tisler ^59^.

Examining region-specific variation in the prevalence of antimicrobial resistance may help identify areas where specific management practices warrant more attention (i.e., ‘hot spots’). For example, in parts of the US, the prevalence of AMR was 64% (*n* = 135 isolates) and 35% (*n* = 135 isolates) in environmental isolates from conventional and organic farms, respectively. Countries with low antimicrobial usage in food production animals, like Sweden and New Zealand, reported low AMR prevalence from environmental samples. For instance, Sweden reported 5% (*n* = 725 isolates) AMR prevalence on both organic and conventional farms, while New Zealand reported that on conventional farms, only 5% of isolates were resistant (*n* = 814 isolates) and 3.8% were resistant (*n* = 814 isolates) on organic farms (Fig. 3).

Patterns for other hosts were also highly variable across broad geographic scales. However, for chickens, the prevalence of AMR was relatively similar across organic and conventional farms (Fig. 3), though patterns from organic farms were particularly notable. In Georgia, USA, the prevalence of AMR in isolates from chickens was marginally higher on organic farms (59%, *n* = 60 isolates) relative to conventional farms (40%, *n* = 60 isolates). In California, USA, the prevalence of AMR in chicken isolates was notably high on both farm types: 78% (*n* = 132 isolates) on conventional farms and 75% (*n* = 132 isolates) on organic farms. These results were primarily driven by drug-resistant *Campylobacter* spp, discussed in more detail below.

### Region-specific patterns in foodborne pathogens

Studies included in our review covered patterns of AMR prevalence in five pathogens sampled from a total of 61,299 isolates: *Escherichia coli*, *Salmonella* spp, *Campylobacter* spp, *Enterococcus,* and *Staphylococcus aureus*. These isolates were sampled from organic farms and conventional farms in Asia (*n* = 1,164 isolates), Europe (*n* = 11,759 isolates), North America (*n* = 44,979 isolates), Oceania (*n* = 3,085 isolates), and South America (*n* = 312 isolates).

### Asia

**Figure 4:**
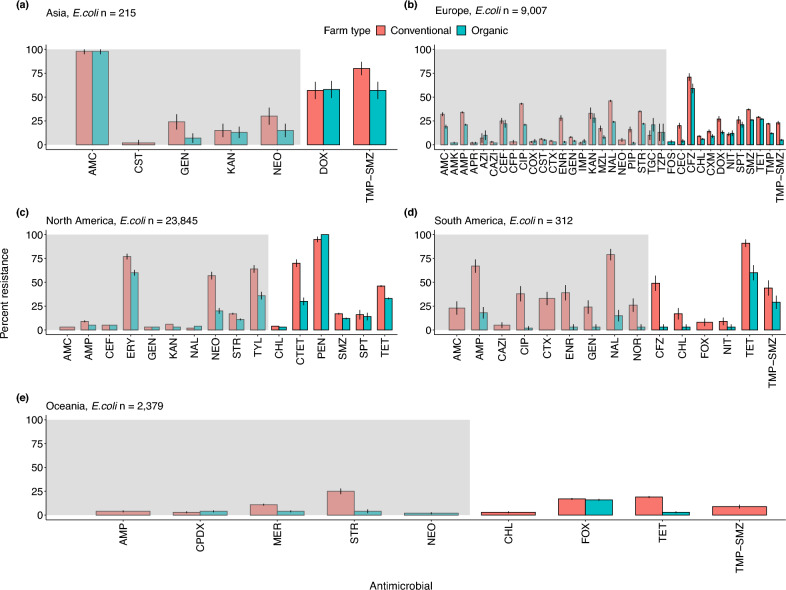
Patterns of antimicrobial resistance in *E. coli.* The AMR prevalence is shown for the number of isolates (*n*) examined on organic and conventional farms in each geographic region. We included studies with at least 10 isolates. (**a**) Asia, *n* = 215, (**b**) Europe, *n*  = 9,007, (**c**) North America, *n*  = 23,845, (**d**) South America, *n*  = 312, and (**e**) Oceania, *n*  = 2,379. Data represent the mean $\pm$ 95% confidence intervals. The grey shading indicates antimicrobials classified as critically important; the unshaded region indicates highly important antimicrobials. For drug acronyms, see Supplementary Table S2.

**Figure 5:**
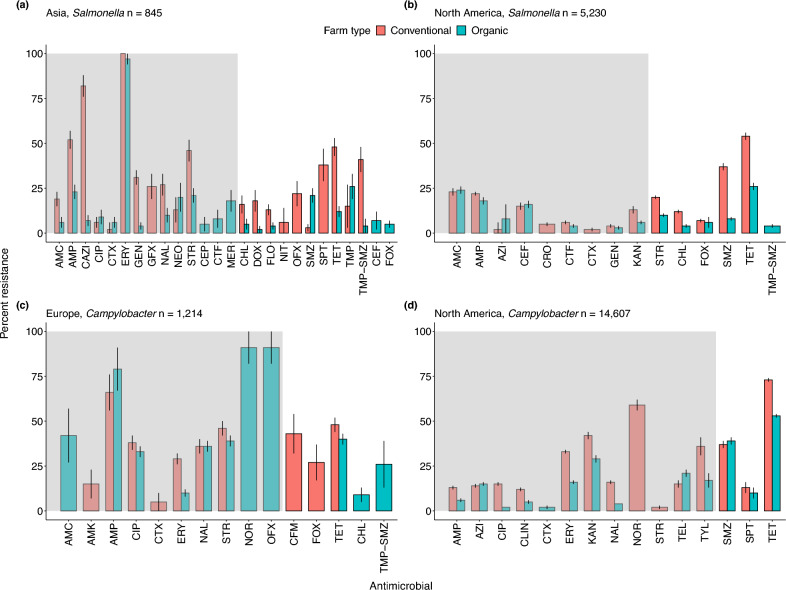
Patterns of antimicrobial resistance in *Salmonella* and *Campylobacter.* The prevalence of antimicrobial resistance is shown for the number of isolates (*n*) examined on organic and conventional farms in each geographic region. We inlcuded studies with at least 10 isolates. (**a**) Asia: *Salmonella*, *n*  = 845, (**b**) North America, *Salmonella,* *n*  = 5,230 (**c**) Europe: *Campylobacter,* *n*  = 1,214, (**d**) North America: *Campylobacter,* *n*  = 14,607. Data represent the mean $\pm$ 95% confidence intervals. The grey shading indicates antimicrobials classified as critically important; the unshaded region indicates highly important antimicrobials. For drug acronyms, see Supplementary Table S2.

**Figure 6:**
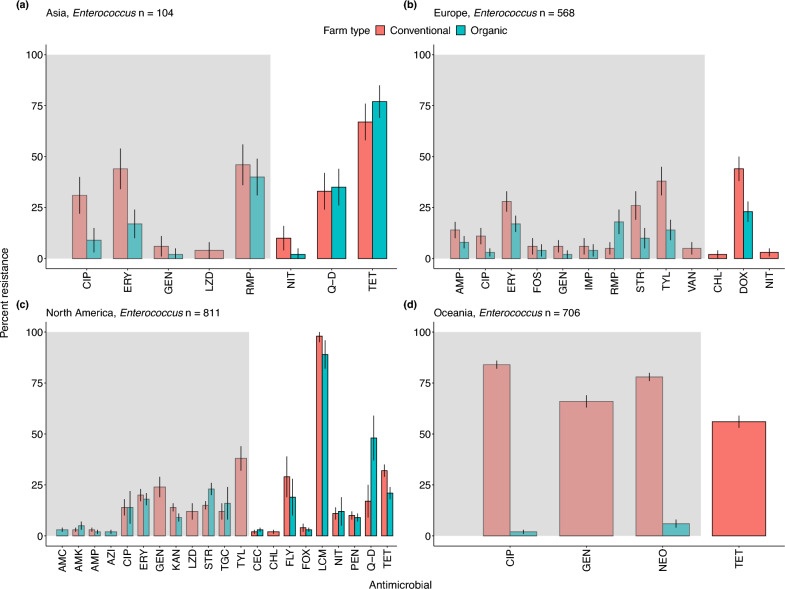
Patterns of antimicrobial resistance in *Enterococcus.* The prevalence of antimicrobial resistance is shown for the number of isolates (*n*) examined on organic and conventional farms in each geographic region. We included studies with at least 10 isolates. (**a**) Asia: *Enterococcus,* *n*  = 104, (**b**) Europe: *n*  = 568, (**c**) North America: *n*  = 811, (**d**) Oceania*:* *n*  = 706. Data represent the mean $\pm$ 95% confidence intervals. The grey shading indicates antimicrobials classified as critically important; the unshaded region indicates highly important antimicrobials. For drug acronyms, see Supplementary Table S2.

Across Asia, the prevalence of AMR was relatively high — and similar — across both organic and conventional farms, regardless of pathogen type. *E. coli* isolates were highly resistant (98%, CI: 95–100%) to amoxicillin-clavulanic acid on both organic (*n* = 105 isolates) and conventional farms (*n* = 110 isolates, Fig. 4a). A similar trend was observed for erythromycin-resistant *Salmonella*: 100% of isolates were resistant (*n* = 171 isolates) on conventional farms and 98% of isolates were resistant (CI: 96–100%, *n* = 165) on organic farms (Fig. 5a). Moreover, *E. coli* and *Salmonella* also showed high resistance to other critically important antimicrobials like erythromycin, regardless of farm management type. In contrast, *Enterococcus* isolates exhibited lower resistance to ciprofloxacin on organic farms, 12% (CI: 10–13%, *n* = 104) versus 48% (CI: 45–53%) on conventional farms (Fig. 6a).

### South America (Brazil)

In South America, conventional farms reported higher resistance in *E. coli* isolates relative to other pathogens. The prevalence of AMR resistance tended to be higher on conventional farms, especially for ampicillin, nalidixic acid, and tetracycline: 67% (CI: 65–73%, *n* = 132 isolates), 79% (CI: 75–84%, *n* = 132 isolates), and 49% (CI: 45–55%, *n* = 132 isolates), respectively as compared to 18% (CI: 15–22%, n = 51 isolates), 32% (CI: 28–35%, *n* = 32 isolates), and 14% (CI: 10–16%, *n* = 14 isolates) on organic farms (Fig. 4d).

### European Union and the United Kingdom

**Figure 7:**
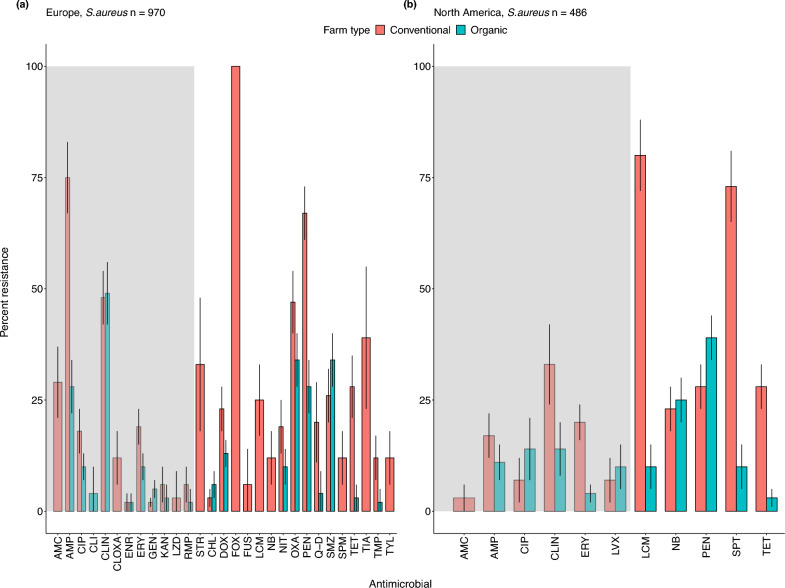
Patterns of antimicrobial resistance in *S. aureus.* The AMR prevalence is shown for the number of isolates (*n*) examined on organic and conventional farms in each geographic region. We included studies with at least 10 isolates. (**a**) Europe: *n*  = 970, (**b**) North America: *n*  = 486. Data represent the mean $\pm$ 95% confidence intervals. The grey shading indicates antimicrobials classified as critically important; the unshaded region indicates highly important antimicrobials. For drug acronyms, see Supplementary Table S2.

Across Europe, and on both conventional and organic farms, we found high levels of resistance to quinolones, like norfloxacin and ofloxacin. These drugs are considered ‘critically important antimicrobials’ and were restricted in 2009 by the EU. For example, the prevalence of resistance in *Campylobacter* was 91% (CI: 82–100%, *n* = 43 isolates) on both conventional and organic farms (Fig. 5c). Notably, however, ampicillin-resistant *Campylobacter* were slightly more prevalent on organic farms (79%, CI: 67–91%, *n* = 43 isolates) relative to conventional farms (66%, CI: 56–76%, *n* = 41 isolates). For *Enterococcus,* 100% of isolates (*n* = 36 isolates) from conventional farms were cefoxitin-resistant (Fig. 7a). In contrast, resistance to erythromycin was relatively low on both conventional and organic farms (6%, CI: 1–8%, *n* = 284 isolates, Fig. 7a). We also found that the prevalence of rifampicin-resistant *Enterococcus* was higher on organic farms (19%, CI: 15–22%, *n* = 134 isolates) as compared to conventional farms (5%, CI: 3–7%, *n* = 134 isolates, Fig. 6b).

### North America

Across North America, *E. coli* showed high levels of resistance to some critically important antimicrobials like erythromycin, and resistance levels were similar on both conventional and organic farms. For example, the prevalence of erythromycin-resistant *E. coli* was 74% (CI: 70–76%, *n* = 789 isolates, Fig. 4c) on conventional farms and 65% (CI: 62–67%, *n* = 789 isolates, Fig. 4c) on organic farms. We also found higher resistance to lincomycin and spectinomycin in *S. aureus* isolates on both conventional farms (74%, CI: 72–77%, *n*  = 554 isolates) and organic farms (68%, CI: 62–74%, *n*  = 418 isolates, Fig. 7b). We found similar trends for penicillin resistance in *E. coli* isolates on both organic farms (100%, *n*  = 2,346 isolates) and conventional farms (96%, CI: 94–98%, *n*  = 3764 isolates). Conversely, the prevalence of AMR in *Salmonella* isolates was relatively low. For example, levels of azithromycin-resistant *Salmonella* were 12% (CI: 8–14%, *n*  = 511 isolates) on organic farms and 10% (CI: 8–12%, *n*  = 511 isolates) on conventional farms. Additionally, levels of gentamicin-resistance were even lower: 3% (CI: 2–4%, *n*  = 118 isolates) on organic farms and 5% (CI: 4–6%, *n*  = 118 isolates) on conventional farms. *Enterococcus* isolates showed high levels of resistance to lincomycin on conventional farms (96%, CI: 92–97%, *n*  = 42 isolates) and 75% on organic farms (CI: 72–78%, *n*  = 42 isolates, Fig. 6c).

### Oceania

Given the extremely low sample sizes, results from Oceania warrant further investigation. We include them here, in part to highlight the paucity of data and the variable levels of resistance. For example, the prevalence of ampicillin-resistant *E. coli* was 4% (CI: 2–6%, *n* = 375 isolates) on conventional farms, while the level of neomycin resistance was 3% (CI: 2–5%, *n* = 375 isolates) on organic farms (Fig. 4e). In contrast, *Enterococcus* isolates showed high levels of resistance to ciprofloxacin (84%, (CI: 82–86%, *n* = 353 isolates), neomycin (74%, CI: 70–73%, *n* = 353 isolates), and gentamicin (67%, CI: 65–69%, *n* = 353 isolates Fig. 6d) on conventional farms. No data for organic farms in this region were available.

## Discussion

Our findings suggest that overall, antimicrobial resistance (AMR) was *slightly* lower in organic livestock production systems relative to their conventional counterparts, while also revealing significant context-dependent variation in this pattern. Specifically, the prevalence of AMR was 18% on organic farms and 28% on conventional farms. However, the substantial region- and country-specific variations in regulations and policies governing organic farming obfuscate broad-scale and generalizable patterns on how organic farming practices affect AMR. Countries have taken markedly different approaches to the guidelines and regulatory agencies that govern the use of antimicrobials in both conventional and organic livestock production (Supplementary Table S1). Further, in practice, these regulations are often overlooked or met with strong resistance at the industry-level.

For example, the European Union (EU) banned the use of antimicrobials for growth promotion (APGs) in livestock production systems in 2006^20^ and the United States followed suit in 2017^21,22^. These policy changes, however, resulted in a ‘repackaging’ of both labeling and marketing practices for these products, characterizing them as ‘prophylactic therapeutics’ instead of ‘growth promoters’^22,23^. Moreover, the US and countries across Europe reported an increasein the use of antimicrobials for prophylactic purposes after the ban of AGPs^6,24^. Thus, these well-intentioned policy changes backfired due, in part, to loopholes that recharacterized the technical nature of the antimicrobials ― a legal but questionable work-around that remains unaddressed.

More stringent regulations on drug use in livestock matter not just for conventional farming but also for organic farming, which seeks to limit but not always eliminate antimicrobial usage in production animals (Supplementary Table S1). In the US, use of antimicrobials is prohibited for organic livestock, while the European regulation for organic dairy herds allows a maximum of three treatments with antimicrobials per cow per year ^25–27^. Denmark, the United Kingdom, and Norway adopted their own regulations, imposing more stringent prohibitions on the use of antimicrobials for growth promotion and requiring supervision by a veterinarian for the use of a limited number of antimicrobials^28,29^. Outside the EU and US, however, conventions governing policies on organic production can be more variable within a country and can become even more challenging to standardize on a national level (Supplementary Table S1). For instance, in Canada, regulations can vary both within and across provinces for products that are distributed and sold solely within those regions^30^.

These differences underscore the need for a more comprehensive and global governance framework to review the science underpinning policies and regulations for both conventional and organic livestock production systems. Such advances are critical to improving investment in research and development to provide viable, non-pharmacological alternatives for disease management in livestock and to move toward global standards, policies, and regulations for livestock production systems. These directives are essential to understanding how, when, or where specific practices of ‘organic’ livestock production reduce the prevalence of AMR. Investment in research and development in these areas is crucial to identifying practical and scalable solutions for farmers who depend on antimicrobials to prevent outbreaks and maintain herd health.

The high levels of drug resistance found in environmental samples further emphasize the need to regulate antimicrobial use and contamination across livestock production systems (Fig. 2c and d). The use of manures from various sources on organic crops differs depending on the specific regulatory framework (e.g., US regulations allow use of manures on organic crops from conventional Confined Animal Feeding Operations (CAFOs), as opposed to EU regulations, which limit the use of manures from “industrial” animal operations). Thus, environmental contamination of antimicrobials may be more prevalent on organic land certified under regulatory frameworks that allow regular use of these industrial manures sourced from conventional farms that routinely use antimicrobials. For example, the use of conventional poultry manure is commonplace in organic grain production in the US, including the production of feed for organic poultry operations. 

While more detailed studies are needed to address the mechanistic underpinnings of these results, at least three key factors could play an important role. First, transition times between a conventional livestock production system that switches to organic management practices and previous land use patterns can substantially impact the levels and diversity of environmental contaminants. The studies included in our review likely differ in the timing of the transition from conventional to organic farming, especially given country- and region-specific variations in regulations on transition time. For instance, the US National Organic Program standards allow for a three-year transition period from conventional to organic management in livestock systems^31,32^, whereas the UK allows a two-year transition period^33^. We were unable to find specific regulations on transition timeframes for other countries.

Transition times matter because animal manure can increase drug residues in the environment, altering the selective pressures that drive antimicrobial resistance. Additionally, contaminated soil can function as reservoir of plasmids (mobile genetic elements) that can transfer resistance within and across species^34,35^. Recent advances in molecular and gene sequencing technologies have increased our awareness that plasmids can transfer among bacteria as well as to other species (e.g., cows to humans), largely through horizontal gene transfer among microbes in the microbiome, leading to rapid transfer of multi-drug resistance in various hosts. This potentiality may increase the risk of antimicrobial resistance ‘spill-over' from soil to livestock, wildlife, and humans^36–39^. Future studies focused on quantitative risk analyses are needed to help identify approaches to mitigate environmental contamination of antimicrobials on both organic and conventional farms. Examining how transition times and soil management practices affect the prevalence of pathogens and AMR is an important first step.

Environmental contamination of antimicrobials may also contribute to the notably high prevalence of AMR on organic poultry farms (Fig. 3). Across some parts of the US, the prevalence of AMR in poultry was slightly higher on organic farms compared to conventional farms. A similar trend was also reported on organic farms in the UK and Portugal, but we are unable to compare the results to conventional farms in these two countries due to lack of data. While organic practices and regulations are, again, highly variable across different regions, access to outdoor grazing may expose chickens to contaminants in the soil, including antimicrobials and microbes or insects, which can serve as reservoirs for drug resistance^40^. Exposure to antimicrobial contaminants in soils may increase the prevalence of AMR in insect reservoirs and thus in poultry.

Our results highlight that drug resistance in *Campylobacter* spp. may warrant particular attention in organic poultry systems. For example, 60–80% of global *Campylobacter* cases originate from poultry products, and 400–500 million cases are reported globally every year^41^. Annually in the US, approximately 310,000 cases of *Campylobacter* are potentially untreatable due to resistance to azithromycin and ciprofloxacin, two important anti-*Campylobacter* antibiotics^42^. For *Campylobacter*, the prevalence of resistance to quinolones was notably high on organic farms in Europe. Yet, quinolones are considered critically important antimicrobials and have been restricted for use in livestock and humans in the EU since 2018^43^. We also found high levels of resistance to drugs considered critically and highly important to human medicine in Asia, Europe, and North America and across a wide gradient of organic and conventional management practices (Fig. 4b, 5a, 5b, 5c, and 5d). Given the public health concerns related to *Campylobacter*, our results join others in calling for greater regulations in these components of livestock management^44,45^.

Our study had several limitations. First, the variation in organic livestock management practices and regulations at national and regional levels strongly limits the generalizability of our results. Second, despite a comprehensive literature search with broad search terms, our study yielded a relatively limited number of studies (*n* = 72) and very few studies from low and middle-income countries (e.g., Africa [*n*  = 0], Oceania [*n*  = 2], and South America [*n*  = 1]). Third, our search criteria only included studies written in English and Portuguese. This language limitation may have caused us to miss studies written in other languages. While we recognize and regret this common limitation, additional language searchers are beyond the scope of this current study. In addition, AMR point prevalence surveys use various methodologies for susceptibility testing and thus results are relative though not quantitative per se. For example, the studies included here that report the prevalence of AMR to spectinomycin and lincomycin used binary metrics (excluding intermediate resistance), which may over- or under-estimate the prevalence of resistance.

Given these limitations, our results should be interpreted with caution as they capture only a small snapshot of the true state of organic farming practices and global patterns of AMR. Indeed, our review and synthesis serve, in large part, to highlight these discrepancies and the paucity of data required to understand links between AMR use in livestock and broad patterns of AMR. The important hypothesis that organic practices for livestock production reduce the prevalence of antimicrobial resistance is often taken at face value. Yet, as we show here, data to address this hypothesis are largely lacking (as evidenced by the small sample size produced from our literature search). Moreover, rigorous and large sample sizes are especially needed to test this hypothesis because relationships between antimicrobial use and drug resistance vary dramatically across contexts, differing between hosts, pathogens, and country-specific regulations (Figs. 1–6). The similarities in patterns of AMR prevalence across broad geographic regions with markedly different practices for regulating drug usage suggest that in some cases organic livestock practices have, at best, marginally reduced the prevalence of AMR. In other cases (e.g., free-range poultry), however, organic farms suffer from a notably high prevalence of AMR that warrants further investigation.

The trends presented here are consistent with previous research indicating high multidrug resistance in *E.coli* and *Salmonella* found in livestock^46,47^. Given a projected 14% increase in consumer demand for meat products by 2030^48^, AMR in livestock will continue to increase unless substantial management changes are implemented. Traditional interventions like stringent cleaning, antibiotics, and vaccines are critical for managing herd health and treating disease. In isolation, however, these costly and reactive approaches aimed at limiting pathogen proliferation can select for more virulent and resistant variants and ultimately ease their spread. The growing threat of antimicrobial resistance and consumer demands to reduce the use of antimicrobials in livestock emphasize the critical need to leverage non-pharmacological approaches to prevent and manage disease^49–53^.

Our results underscore the need for multidisciplinary and global approaches that blend organic farming principles and non-pharmacological interventions to reduce routine antibiotic use in livestock. In addition, research on interventions like bacteriophages, probiotics, and increased surveillance of antimicrobial resistance have shown promising results in reducing AMR^1,54^. Furthermore, collaborations among stakeholders (i.e., farmers, researchers, and policymakers in the animal health sector) could help disseminate information and best practices. Unfortunately, the industry continues to move in the opposite direction, particularly with the rapidly expanding trend toward growth of corporate-owned livestock farms that control both the dietary and pharmaceutical regimes of the animals^55^. The lack of regulations and transparency in these practices prevent a clear understanding of when and in what quantities antibiotics are provided in feed, for instance.

Future studies could help formulate scalable solutions for conventional farming practices with benefits for both agriculture and public health. Our review indicates that a key focal area includes a better understanding of how transition times and soil properties influence the prevalence, viability, and retention of pathogens — and the genes that harbor AMR^55,56^. Taken together, these results emphasize the inherent challenges to understanding links between drug use, livestock production practices, and drug resistance. Greater understanding of how, when, and where antimicrobials can be reduced in livestock production systems (e.g., by adopting a subset of select organic-based practices) without a concomitant increase in disease outbreaks would greatly enhance efforts to reduce the evolution of drug resistance and extend the 'shelf life' of these powerful biomedical tools. As our synthesis highlights, we are far from reaching such an understanding. We hope that by synthesizing these challenges, our study catalyzes future empirical research to address these gaps in knowledge.

## Methods

### Literature search strategy

Our initial goal was to examine studies that directly compared patterns of antibiotic resistance from organic and conventional farms within the same region (i.e., US: state; Africa: province; Canada: province; UK: county) and livestock species (e.g., cow, chicken). However, these initial search criteria were too restrictive and yielded only sixty-four studies. Therefore, we expanded the search to include studies that reported antimicrobial resistance from organic farms without always directly comparing their patterns alongside conventional counterparts.

We conducted literature searches for studies published between 2000 and 2022 using three electronic databases (PubMed, Web of Science, and PubAg). We used the following search terms, which we modified slightly for each database. Note, for brevity, we show abbreviated terms (e.g., “livestock names” reflects individual searches for sheep, goats, chickens, etc., and the name of the pathogen reflects individual searches for each pathogen ‒ i.e., *Campylobacter*, *E. coli*, *Salmonella,* etc. In the PubMed search, for example, we used the following terms: livestock name AND product OR livestock production OR livestock farm OR name of pathogen OR antimicrobial AND resistant OR agriculture OR conventional OR organic AND agriculture. The full search terms are provided in the supplementary methods S1. Finally, references from other literature reviews ^42,42^ and all other studies were screened for inclusion. Our search generated 1,836 studies for the initial screening (Fig. 1a).

### Study selection

We reviewed all English-language and Portuguese-language articles that directly compared patterns of antimicrobial resistance (AMR) from chicken, turkey, cattle, pigs, and environmental samples from organic and conventional farms in a given geographic region. After screening the reference lists, we excluded reviews, unrelated topics, and book chapters (Fig. 1a). Following the search, all records were exported to Endnote’s web citation manager^57^, and duplicates were removed. The records were then exported to a spreadsheet and organized by title, doi, authors, journal, year of publication, and abstract. Finally, the titles and abstracts were screened against the inclusion criteria.

The studies that met the eligibility criteria were retrieved in full text and were thoroughly reviewed. Seventy-two studies met our inclusion criteria (Fig. 1a). We attributed the reduction in sample size to two conditions: (1) our search terms covered general antimicrobial resistance and antimicrobial susceptibility topics and (2) our search focused only on articles written in English and Portuguese. As a way of assessing data quality in our review, we excluded records that did not clearly identify farm types as organic or conventional, gave no geographic information on study location, provided unclear resistance rates, or involved imported products. Data extraction results were stratified according to country name, antimicrobial resistance results, farm type, and pathogens.

### Statistical analysis

All data analyses were conducted in R version 4.2.0^58^ and QGIS version 3.24.0-Tisler^59^. To examine differences in the prevalence of AMR on organic and conventional farms, we used generalized linear models (GLMs) with quasibinomial distributions and log link functions^60^. We built candidate models starting with the full model with all combinations of main effects among relevant biological and methodological factors, while avoiding overfitting. Specifically, to examine overall changes in the prevalence of AMR across the 19-year time frame included in this review, the full model included farm type (organic vs. conventional), country, study year, and their interaction as fixed effects. Then, to examine more fine-scale differences in the prevalence of AMR, the full model examined effects of farm type, host, pathogen, country, and their interactions. We excluded antimicrobial type because the large number of drugs covered in these studies led to overfitting the models.

Following Burnham and Anderson, we compared candidate models using Akaike’s information criterion and ΔAIC (the difference in AIC values for the focal model and the model with the lowest AIC, i.e., the ‘winning’ model)^61^. We conducted model selection analyses using the *aictab* function in the R package AICcmodavg^62^. We also calculated the Akaike weight (*ω*), which further quantifies the probability that a model is the most appropriate model relative to the candidate models. ΔAIC less than two and a higher (*ω*) generally indicates that a model has substantial support while a suite of best models with low weights (*ω* ~ 0) indicates that no single variable plays a substantial role in mediating patterns of AMR^61^.

Using the *Anova* function in the R package car^63^, we assessed significance using Wald χ 2 statistics for the winning model. We also evaluated model fits with visual diagnostics, quantile–quantile plots, and residual-versus-predictor plots^64,65^. Note, the MIC (mean inhibitory concentration) values and MIC breakpoints were not used in this analysis; variations in methodologies and criteria used across laboratories and differing epidemiological contexts pose substantial challenges for standardization (in addition the other challenges outlined in the Discussion). Therefore, we focused on estimates of the prevalence of AMR as a relatively consistent measure of resistance across studies. To report the prevalence of AMR in foodborne pathogens, we calculated the pooled prevalence of resistance from each pathogen-drug combination^5,66^ using the formula below:

$$\mathrm{Pooled}\,\mathrm{prevalence}=\sum \frac{\mathrm{Number}\,\mathrm{of}\,\mathrm{isolates}\,\mathrm{resistant}}{\mathrm{Total}\,\mathrm{number}\,\mathrm{of}\,\mathrm{isolates}\,\mathrm{tested}}$$

## Data Availability

All data generated or analyzed from this study, as well as Supplementary Information, are available in Zenodo public repository: https://zenodo.org/record/7600391#.Y9v6TezMK3I^67^.
